# The GPR 55 agonist, L-α-lysophosphatidylinositol, mediates ovarian carcinoma cell-induced angiogenesis

**DOI:** 10.1111/bph.13196

**Published:** 2015-06-26

**Authors:** Nicole A Hofmann, Jiang Yang, Sunia A Trauger, Hironao Nakayama, Lan Huang, Dirk Strunk, Marsha A Moses, Michael Klagsbrun, Joyce Bischoff, Wolfgang F Graier

**Affiliations:** 1 Institute for Molecular Biology and Biochemistry, Medical University GrazGraz, Austria; 2 Vascular Biology Program, Boston Children's HospitalBoston, MA, USA; 3 Department of Surgery, Harvard Medical SchoolBoston, MA, USA; 4 Department of Pathology, Harvard Medical SchoolBoston, MA, USA; 5 FAS Small Molecule Mass Spectrometry Facility, Harvard UniversityBoston, MA, USA; 6 Experimental and Clinical Cell Therapy Institute, Paracelsus Medical UniversitySalzburg, Austria

## Abstract

**Background and Purpose:**

Highly vascularized ovarian carcinoma secretes the putative endocannabinoid and GPR55 agonist, L-α-lysophosphatidylinositol (LPI), into the circulation. We aimed to assess the involvement of this agonist and its receptor in ovarian cancer angiogenesis.

**Experimental Approach:**

Secretion of LPI by three ovarian cancer cell lines (OVCAR-3, OVCAR-5 and COV-362) was tested by mass spectrometry. Involvement of cancer cell-derived LPI on angiogenesis was tested in the *in vivo* chicken chorioallantoic membrane (CAM) assay along with the assessment of the effect of LPI on proliferation, network formation, and migration of neonatal and adult human endothelial colony-forming cells (ECFCs). Engagement of GPR55 was verified by using its pharmacological inhibitor CID16020046 and diminution of GPR55 expression by four different target-specific siRNAs. To study underlying signal transduction, Western blot analysis was performed.

**Key Results:**

Ovarian carcinoma cell-derived LPI stimulated angiogenesis in the CAM assay. Applied LPI stimulated proliferation, network formation, and migration of neonatal ECFCs *in vitro* and angiogenesis in the *in vivo* CAM. The pharmacological GPR55 inhibitor CID16020046 inhibited LPI-stimulated ECFC proliferation, network formation and migration *in vitro* as well as ovarian carcinoma cell- and LPI-induced angiogenesis *in vivo*. Four target-specific siRNAs against GPR55 prevented these effects of LPI on angiogenesis. These pro-angiogenic effects of LPI were transduced by GPR55-dependent phosphorylation of ERK1/2 and p38 kinase.

**Conclusions and Implications:**

We conclude that inhibiting the pro-angiogenic LPI/GPR55 pathway appears a promising target against angiogenesis in ovarian carcinoma.

## Tables of Links

**Table:** 

TARGETS
**GPCRs***^a^*2013b
GPR55
**Enzymes***^b^*2013b
ERK1/2
p38

**Table:** 

LIGANDS
AM251
CID16020046
LPI, L-α-lysophosphatidylinositol
SR144528
U0126

These Tables list key protein targets and ligands in this article which are hyperlinked to corresponding entries in http://www.guidetopharmacology.org, the common portal for data from the IUPHAR/BPS Guide to PHARMACOLOGY (Pawson *et al*., 2014) and are permanently archived in the Concise Guide to PHARMACOLOGY 2013/14 (*^a,b^*Alexander *et al*., 2013a,b[Bibr b1],[Bibr b2]).

## Introduction

Ovarian cancer is the most common cause of death from gynaecological cancers (Siegel *et al*., 2012) and a high level of angiogenesis is a poor prognostic marker in ovarian carcinoma patients (Schoell *et al*., 1997; Banerjee and Kaye, 2011). Clinical studies have revealed that patients with ovarian and peritoneal cancer show elevated levels of lysophospholipids in blood and ascites fluids (Xiao *et al*., 2000; 2001[Bibr b46],[Bibr b47]; Xu *et al*., 2001; Murph *et al*., 2007), suggesting that lysophospholipids might be a biomarker for these highly vascularized tumours (Sutphen *et al*., 2004; Murph *et al*., 2007; Pineiro and Falasca, 2012). Recently, it was shown that L-α-lysophosphatidylinositol (LPI), but not other lysophospholipids, secreted by ovarian and prostate carcinomas regulated cancer cell growth via an autocrine loop (Pineiro *et al*., 2011). However, the potential roles of LPI in tumour angiogenesis have not been well explored.

LPI has been shown to be produced and secreted by various cell types, including human platelets (Billah and Lapetina, 1982), endothelial cells (Hong and Deykin, 1982; Martin and Wysolmerski, 1987; Bondarenko *et al*., 2010) and peripheral blood (PB) neutrophils (Smith and Waite, 1992), as well as cancer cells (Pineiro *et al*., 2011). Further studies have revealed various physiological and pathophysiological functions related to LPI, including insulin release by pancreatic cells, pain, obesity/type 2 diabetes, bone resorption and cancer (Ford *et al*., 2010; Pineiro and Falasca, 2012). However, the lack of a specific LPI receptor held back scientific research and the development of targeted therapies.

In 2007, Oka *et al*. (2007) showed that LPI was a specific agonist for the orphan GPCR, GPR55, first cloned in 1999 (Sawzdargo *et al*., 1999). Crystallographic analysis showed that GPR55 consists of seven transmembrane α helices in which LPI binds among the transmembrane helices 2, 3, 6 and 7 with the highest interaction energy as compared with other tested GPR55 agonists (Kotsikorou *et al*., 2011). The GPR55 was further shown to be sensitive, although to a lesser extent, to the endocannabinoid anandamide which suggested GPR55 might be a putative cannabinoid (Waldeck-Weiermair *et al*., 2008; Zhang *et al*., 2010). The discovery of a receptor-mediated biological action of LPI has allowed new investigations on the physiological and pathological functions of this bioactive lysophospholipid (Pineiro and Falasca, 2012; Liu *et al*., 2015). It is well established that following binding of LPI to GPR55, intracellular Ca^2+^ mobilization is increased (Waldeck-Weiermair *et al*., 2008; Bondarenko *et al*., 2010; 2011a,b[Bibr b8],[Bibr b9]; Oka *et al*., 2010) and several signalling cascades are increased, including ERK1/2 (Oka *et al*., 2007; Whyte *et al*., 2009; Andradas *et al*., 2011; Pineiro *et al*., 2011), RhoA (Henstridge *et al*., 2009; Kargl *et al*., 2013) and MAPK p38 (Oka *et al*., 2010) pathways. Furthermore, LPI sustains depolarization of membranes through inhibition of Na^+^/K^−^-ATPase and activation of non-selective cation channels (Bondarenko *et al*., 2010; 2011a,b[Bibr b8],[Bibr b9]). In endothelial cells, the LPI/GPR55 axis has been shown to (i) increase proliferation (Zhang *et al*., 2010); (ii) influence motility (Murugesan and Fox, 1996; Kargl *et al*., 2013); and (iii) induce expression of adhesion molecules (VCAM-1 and ICAM1) (Kume *et al*., 1992). Recently, the compound CID16020046 was shown to be a selective and efficient antagonist for GPR55, but not for other cannabinoid receptors, including CB_1_ and CB_2_ receptors (Kargl *et al*., 2013).

The ability of tumours to secrete growth factors and induce new blood vessel formation has become a central focus in cancer research (Potente *et al*., 2011). Although various growth factors including VEGF and basic fibroblast growth factor (bFGF) have been shown to play a major role in angiogenesis, other factors (e.g. angiopoietins and hepatocyte growth factor) are also involved (Welti *et al*., 2013). For decades, isolated endothelial cells have served as model system to study the effect of growth factors and inhibitors (Basile and Yoder, 2014). Primary human endothelial colony-forming cells (ECFCs) are a subpopulation of endothelial progenitor cells (Yoder *et al*., 2007) and are considered a reliable endothelial/angiogenic model due to their high proliferative potential and robust vessel formation *in vivo* (Yoder *et al*., 2007; Melero-Martin *et al*., 2008; Reinisch *et al*., 2009; Hofmann *et al*., 2012).

Based on reports that LPI is produced and secreted by highly vascularized ovarian carcinomas (Pineiro *et al*., 2011; Pineiro and Falasca, 2012), we decided to investigate the potential role of the LPI/GPR55 axis in promoting angiogenesis. Accordingly, we investigated whether LPI secreted by ovarian carcinoma cells could be a cause of the increased (tumour) angiogenesis. Furthermore, we aimed to determine the effect of LPI/GPR55 on endothelial cell proliferation, network formation, and migration *in vitro* and angiogenesis in an *in vivo* chicken chorioallantoic membrane (CAM) assay as well as the underlying mechanisms. Targeting the LPI/GPR55 axis could represent potential models of pro- and anti-angiogenic treatment.

## Methods

### Cell culture

Human ECFCs were isolated from neonatal cord and peripheral blood and their distinct endothelial phenotypes were verified by flow cytometry as previously described (see Supporting Information Fig. S1) (Hofmann *et al*., 2009; 2012[Bibr b16],[Bibr b17]; Reinisch and Strunk, 2009; Reinisch *et al*., 2009). HUVECs were obtained from Lonza (Basel, Switzerland). ECFC and HUVECs were grown in endothelial growth medium-2 (EGM-2) (Lonza) containing 2% FBS and 1% penicillin/streptomycin/L-glutamine/heparin (Life Technologies, Carlsbad, CA, USA) and EGM-2 growth factor supplements (composed of bFGF, IGF-2, EGF, VEGF, ascorbic acid, hydrocortisone). Ovarian carcinoma cell lines OVCAR-3 (American Type Culture Collection, Manassas, VA, USA), OVCAR-5 (kindly provided by the Cell Culture Core, Vascular Biology Program, Boston Children's Hospital, Boston, MA, USA) and COV-362 (Sigma Aldrich, St. Louis, MO, USA) were grown in DMEM containing 10% FBS.

### Ethics statement

Prior approval was obtained for human cell and tissue sample collection from the Institutional Review Board of the Medical University of Graz (protocols 19-252 ex 07/08, 18-243 ex 06/07, 21.060 ex 09/10). Adult samples were collected after written informed consent from healthy volunteers, and umbilical cord samples after written informed consent by the mother after full-term pregnancies in accordance with the Declaration of Helsinki.

### Extraction of lipids

Up to 19 mL of conditioned medium from 6–10 million cells of OVCAR-3, OVCAR-5, DMEM and 14 mL from COV-362 cells were extracted with 40 mL of acidified 2:1 methanol : chloroform and 0.05 N HCl in a 60 mL separator funnel. The bottom layer was collected and dried under a gentle stream of nitrogen in a 20 mL glass vial. The lipid extract was reconstituted in 200 μL chloroform : methanol (2:1 v/v) prior to injection.

### LPI measurement

Chemical standards of LPI were obtained from Sigma Aldrich. An LC-MS/MS method was optimized on an Agilent (Agilent Technologies, Santa Clara, CA, USA) 6460 triple-quadrupole mass spectrometer using multiple reaction monitoring (MRM) in negative ion mode. Specifically, the MRM transitions used for LPI were 571.3 – >255.1 m/z for quantification and 571.3 – >152.9 m/z for confirmation. A collision energy of 41 V and a fragmentor setting of 207 V were used to monitor both MRM transitions. The most abundant fragment corresponds to the loss of palmitic acid and the secondary fragment corresponds to the subsequent loss of the inositol, leaving a C_3_H_6_O_5_P^−^ ion at 152.9 m/z. Mass spectrometer parameter settings were gas temperature (350°C), gas flow (10 L·min^−1^), nebulizer (30 psi), sheath gas temperature (400°C), sheath gas flow (11 L·min^−1^), capillary voltage (3800 V) and nozzle voltage (500 V). Liquid chromatography conditions with a Dikma-Biobond C4 column 4.6 × 50 mm 5 μm particle size were used for separation. Chromatography method included gradient elution at 0.400 mL·min^−1^ with solvent 20 mM ammonium carbonate/0.1% ammonium hydroxide as mobile phase A and acetonitrile for mobile phase B. The gradient started at 0% B and progressed to 100% A in 16 min, and then changed back to 0% B over 0.1 min, and re-equilibrated for 3.9 min before the next injection. A 10 μL sample injection was used for all standards and samples. An external standard curve was used to calculate concentrations of LPI in different samples between 0.0025 and 0.25 μM. The lower limit of detection and quantification was determined to be at 2.5 nM with a S/N > 12. A validation of the method was done using 20 nM LPI added to DMEM containing 10% FBS and extracted using the method described, and reconstituted in 200 μL chloroform : methanol (2:1 v/v) prior to injection. The control experiment was done using DMEM containing 10% FBS prepared in the same manner. A linear calibration curve was measured for LPI with an *R*^2^ of 0.95.

### Proliferation assay

ECFCs from three different cord blood donors, HUVECs and peripheral blood ECFC were seeded in 24-well plates (Nalge Nunc, Rochester, NY, USA) in EGM-2 at a density of 3000 cells/cm^2^ and allowed to adhere for 24 h. Subsequently, cells were subjected to growth factor-reduced medium [EBM-2 (Lonza) containing 2% FBS and 1% penicillin/streptomycin/L-glutamine/heparin (Life Technologies) without the addition of EGM-2 growth factor supplements] with or without different concentrations of LPI (Sigma) and/or endocannabinoid receptor antagonists: CID16020046 (Tocris Bioscience, Northpoint, Avonmouth, Bristol, UK) and AM251, SR144528 (both Cayman Chemical Europe, Tallinn, Estonia). A 30 min treatment with 10 μM U0126 (Cell Signaling, Cambridge, UK) was also tested. After 48 h, treated cells were harvested and the cell number was counted by a Casy cell counter (Roche, Mannheim, Germany). Nine independent experiments per group were performed in triplicate.

### Matrigel angiogenesis assay

Capillary-like network formation of ECFC, isolated from three different donors, plated on growth factor-reduced Matrigel® (BD, Biosciences, San Jose, CA, USA) was performed according to the instruction manual included in the purchase of Matrigel. The influence of LPI and different endocannabinoid receptor antagonists was tested in growth factor-reduced medium [EBM-2 (Lonza) containing 2% FBS and 1% penicillin/streptomycin/L-glutamine/heparin (Life Technologies) without the addition of EGM-2 growth factor supplements]. Network formation (14–16 h) was documented with a Nikon SPOT camera on a Nikon microscope (Nikon, Amsterdam, The Netherlands). Branch points were counted after 16 h by ImageJ [National Institutes of Health (NIH), Bethesda, MD, USA]. Nine independent experiments per group were performed in triplicate.

### Ovarian cancer cell conditioned media

Ovarian cancer cells OVCAR-3, OVCAR-5 and COV-362 cells were grown in a 10 cm dish with DMEM and 10% FBS until approximately 80% confluent. After washing with PBS, cells were incubated in 10 mL phenol-free DMEM without serum for 24 h. Conditioned medium was collected, centrifuged and immediately used. Non-conditioned phenol red-free DMEM served as negative control.

### CAM assay

Chicken eggs were purchased from Charles River Laboratories (Wilmington, MA, USA) and placed in an incubator at 37°C, 40% humidity. On day 3, up to 8 mL of albumin was aspirated from a small hole made at the bottom of the egg and the hole was sealed with candle wax. Then an approximately 2 cm large window was cracked into the rounded part of the upright egg using Dumont tweezers (6) and the egg membrane was completely removed. The window was covered with a cap of sterilized aluminium foil. The eggs were then incubated in a cell culture incubator at 37°C, 40% humidity, 3% CO_2_. On day 7, up to 2 mm filter paper patches were punched out of sterilized Whatman-filter papers (Sigma Aldrich) and placed on a sterile surface. Five microlitres of treatment solution, control medium or ovarian cancer cell conditioned medium from OVCAR-3, OVCAR-5 or COV-362 cells, as indicated, were dropped on each filter paper allowing it to dry for 15 min and carefully placed on the developing CAM. Blood vessel development was observed daily and pictures were taken 3 days (day 10 of egg development) after treatment with a stereo microscope. Vessels crossing the outline of the filter paper were analysed using ImageJ (NIH). Six to nine independent experiments per group were performed in triplicate.

### Reduction of GPR55 gene expression by siRNA

Transfection of ECFCs with a pool of four validated target-specific GPR55 siRNAs (FlexiTube siRNA; Qiagen, Venlo, The Netherlands) (referred to as siGPR55) or scrambled siRNA (Qiagen) (referred to as sicontrol) was performed using Lipofectamine® RNAiMAX reagent (Invitrogen, Carlsbad, CA, USA) according to the manufacturer's protocol. All experiments were performed 36–48 h after transfection. The efficiency of siRNAs and of an appropriate negative control was determined by Western blot.

### Proteome profiler arrays

The Proteome Profiler Human Phospho-Kinase Array Kit (Cat. No: ARY003B) and Human Angiogenesis Array Kit (Cat. No: ARY007) were obtained from R&D Systems (Minneapolis, MN, USA). ECFCs were treated with vehicle or LPI (10 μM) for 15 min or 24 h, respectively, and analysed according to manufacturer's instructions. The average signal (pixel density) of duplicate spots representing each protein was evaluated by ImageJ (NIH). After background subtraction, a twofold increase was considered to be significant.

### Western blot analysis

ECFCs were serum starved for 6–24 h and subsequently treated with vehicle, LPI and/or CID366791 for 0–60 min. In order to extract the sturdily membrane-bound GPR55, cells were lysed directly with 1× reducing Laemmli (SDS sample) buffer (Boston BioProducts, Inc., Boston, MA, USA) and precipitated 10 min at 90°C. Otherwise, cells were lysed and Western blots were performed as previously described by us (Hofmann *et al*., 2012; 2014[Bibr b17],[Bibr b15]). Specific proteins were detected using antibodies against GPR55 (Thermo Scientific, Tewksbury, MA, USA) and total or phosphorylated ERK1/2 or p38 (all obtained from Cell Signaling), and compared with housekeeping protein control β-actin (Santa Cruz, Dallas, TX, USA). Pixel intensity was determined using ImageJ (NIH). Six independent experiments per group were performed in triplicate.

### Data analysis

‘*n*’ values refer to the number of individual experiments performed. Data were compared using anova and subsequent Bonferroni *post hoc* test or two-tailed Student's *t*-test assuming unequal variances, where applicable. Statistical significance was assumed at *P* < 0.05. EC_50_ and IC_50_ values were calculated out of at least three independent experiments with three to five repeats for each concentration using GraphPad Prism® 5.0f (GraphPad Software, La Jolla, CA, USA) and expressed with the 95% confidence interval provided in parenthesis.

## Results

### Ovarian cancer cells produce LPI and mediate angiogenesis through GPR55

**Figure 1:**
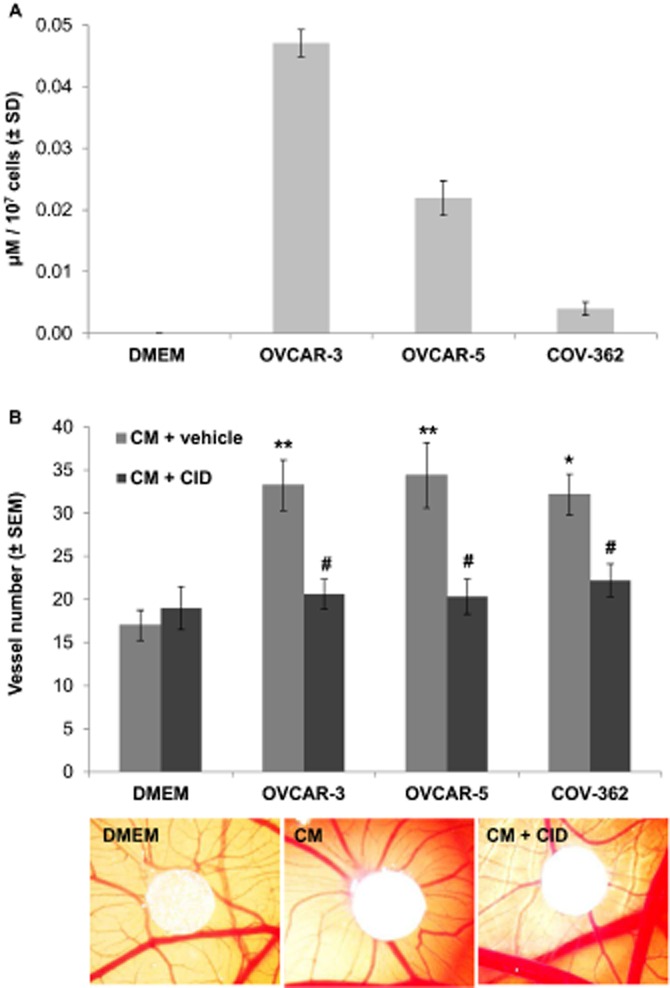
Ovarian cancer cells produce LPI and induce chicken CAM angiogenesis in a GPR55-dependent manner. (A) Quantification of LPI in conditioned medium from three different ovarian cancer cell lines (OVCAR-3, OVCAR-5, COV-362). (B) Quantification of vessel numbers around white filter paper in an *in vivo* CAM assay (by ImageJ). Filter papers were loaded with unconditioned DMEM or 24 h conditioned DMEM (CM) of three different ovarian cancer cell lines (OVCAR-3, OVCAR-5, COV-362), respectively, with or without vehicle or GPR55 inhibitor CID16020046 (CID). Representative macroscopic pictures of CAM angiogenesis around filter paper containing control DMEM, OVCAR-5 CM or OVCAR-5 CM with CID. *n* = 6–9; *P < 0.05; **P < 0.01, significantly different from vehicle control; ^#^*P* < 0.01, significantly different from corresponding ovarian cancer CM. anova followed by Bonferroni test.

Increased serum levels of the GPR55-ligand LPI have been found in patients with high-grade ovarian carcinoma (Xiao *et al*., 2000; 2001[Bibr b46],[Bibr b47]; Xu *et al*., 2001; Sutphen *et al*., 2004; Murph *et al*., 2007; Pineiro *et al*., 2011; Pineiro and Falasca, 2012). To test our hypothesis, that ovarian cancer cells secrete LPI, and thus promote tumour angiogenesis *in vivo* via an LPI/GPR55-dependent mechanism; conditioned medium from the human ovarian cancer cell lines OVCAR-3, OVCAR-5 and COV-362 was analysed for its LPI levels and in the CAM angiogenesis model. LC-MS/MS revealed that OVCAR-3, OVCAR-5 and COV-362 cells produced significant but quite different amounts of LPI (Figure 1A). Within 3 days, conditioned medium from OVCAR-3, OVCAR-5 and COV-362 strongly induced angiogenesis *in vivo* to a similar extent (90–100% increase), compared with unconditioned medium (Figure 1B). Selective inhibition of the LPI receptor GPR55 with CID16020046 (20 μM) effectively blocked ovarian cancer-induced angiogenesis of all tested cell lines (Figure 1B). Together, these results suggest that LPI produced by ovarian cancer cells induces angiogenesis in a GPR55-dependent manner.

### LPI regulates angiogenic potential of endothelial cells *in vitro* and angiogenesis *in vivo*

**Figure 2:**
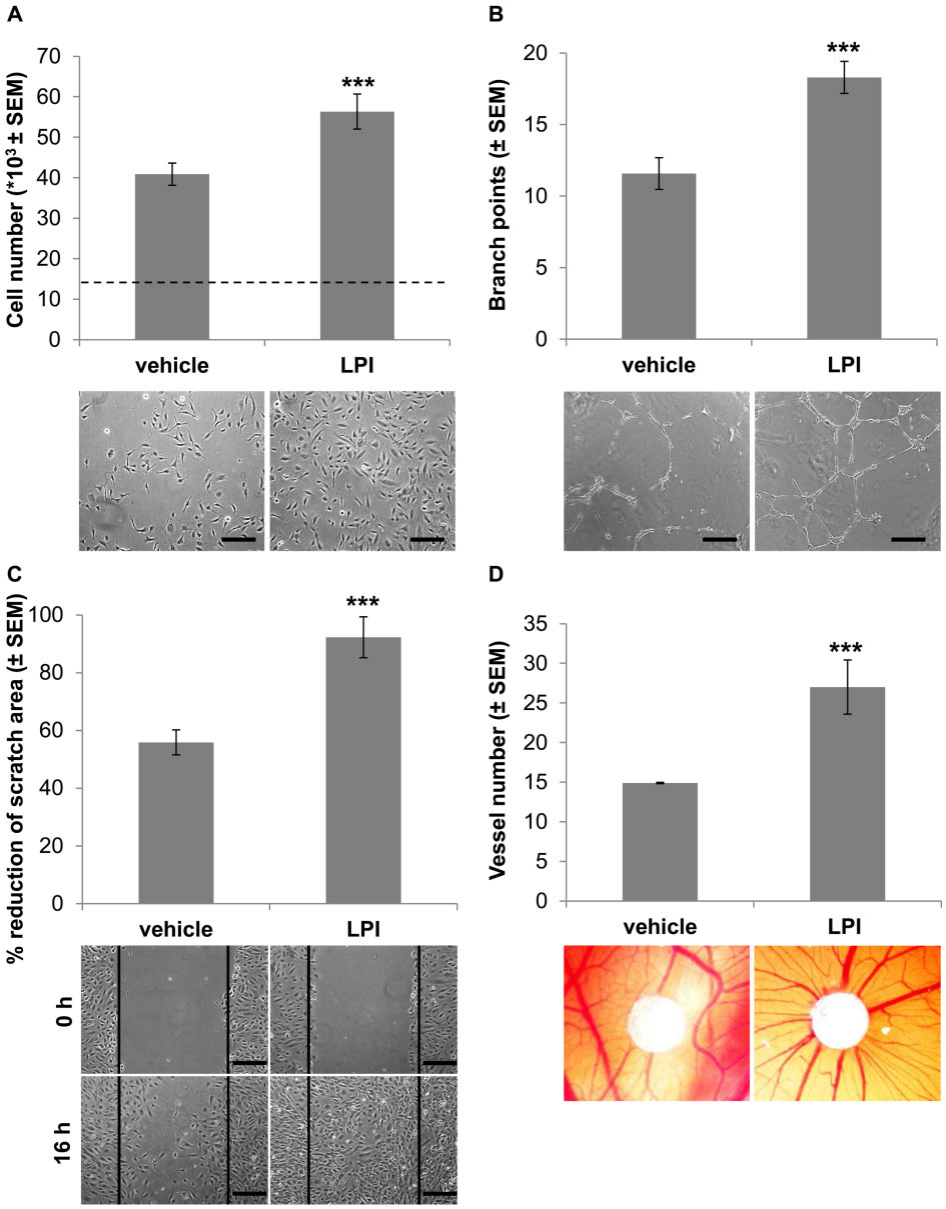
LPI stimulates angiogenesis *in vitro* and *in vivo*. (A–C) Effect of vehicle or 10 μM LPI on neonatal ECFC. (A) Cell numbers (×10^3^) after 48 h *in vitro* proliferation assay. Dotted line marks starting cell number (12.000 cells). (B) Branch point formation in an *in vitro* angiogenesis assay after 16 h. (C) Closure of *in vitro* endothelial scratch area after 16 h. (A–C) Respective representative cell culture pictures with black bars marking 200 μm. *n* = 9; (D) Quantification of vessel numbers around white filter paper in an *in vivo* chicken CAM assay after 72 h with respective representative macroscopic pictures. *n* = 6–9; ***P < 0.001, significantly different from vehicle; Student's *t*-test.

The effects of purified LPI on endothelial cell proliferation, network formation and migration were tested *in vitro* on isolated endothelial colony-forming progenitor cells (ECFCs) derived from three different donors. The isolated human neonatal cord ECFCs showed a distinct endothelial phenotype as shown by expression of typical endothelial cell surface markers (Supporting Information Fig. S1), as previously shown (Hofmann *et al*., 2009; 2012[Bibr b16],[Bibr b17]; Reinisch and Strunk, 2009; Reinisch *et al*., 2009). LPI stimulated ECFC proliferation in a dose-dependent manner with an EC_50_ of 2.8 (2.2–3.6) μM (Supporting Information Fig. S2a). Low concentrations of LPI, resembling the endogenous LPI levels secreted by 10^7^ ovarian carcinoma cells (1 nM), were sufficient to stimulate proliferation of ECFCs (Supporting Information Fig. S2a). The maximum proliferative increase (1.55 ± 0.1-fold increase) was measured within 48 h upon applying 10 μM LPI as compared with vehicle controls (Figure 2A and Supporting Information Fig. S2a) and further experiments were performed at this 10 μM concentration. HUVECs showed a similar increase in proliferation as did isolated human adult peripheral blood ECFCs (Supporting Information Fig. S2b). Furthermore, compared with vehicle controls, 10 μM LPI significantly increased ECFC network formation in an *in vitro* Matrigel assay (Figure 2B) and closure of an endothelial wound in an *in vitro* scratch assay (Figure 2C).

To investigate whether these stimulatory effects could occur *in vivo*, we analysed angiogenesis in a CAM assay. For this purpose, we placed a filter paper soaked and then dried with either vehicle or 10 μM LPI on the developing 7-day-old CAM. Within 72 h, 10 μM LPI had significantly increased vessel formation, compared with vehicle control (Figure 2D). Together, these *in vitro* and *in vivo* results indicate that LPI is a potent pro-angiogenic factor.

### LPI-induced angiogenesis is GPR55 dependent

**Figure 3:**
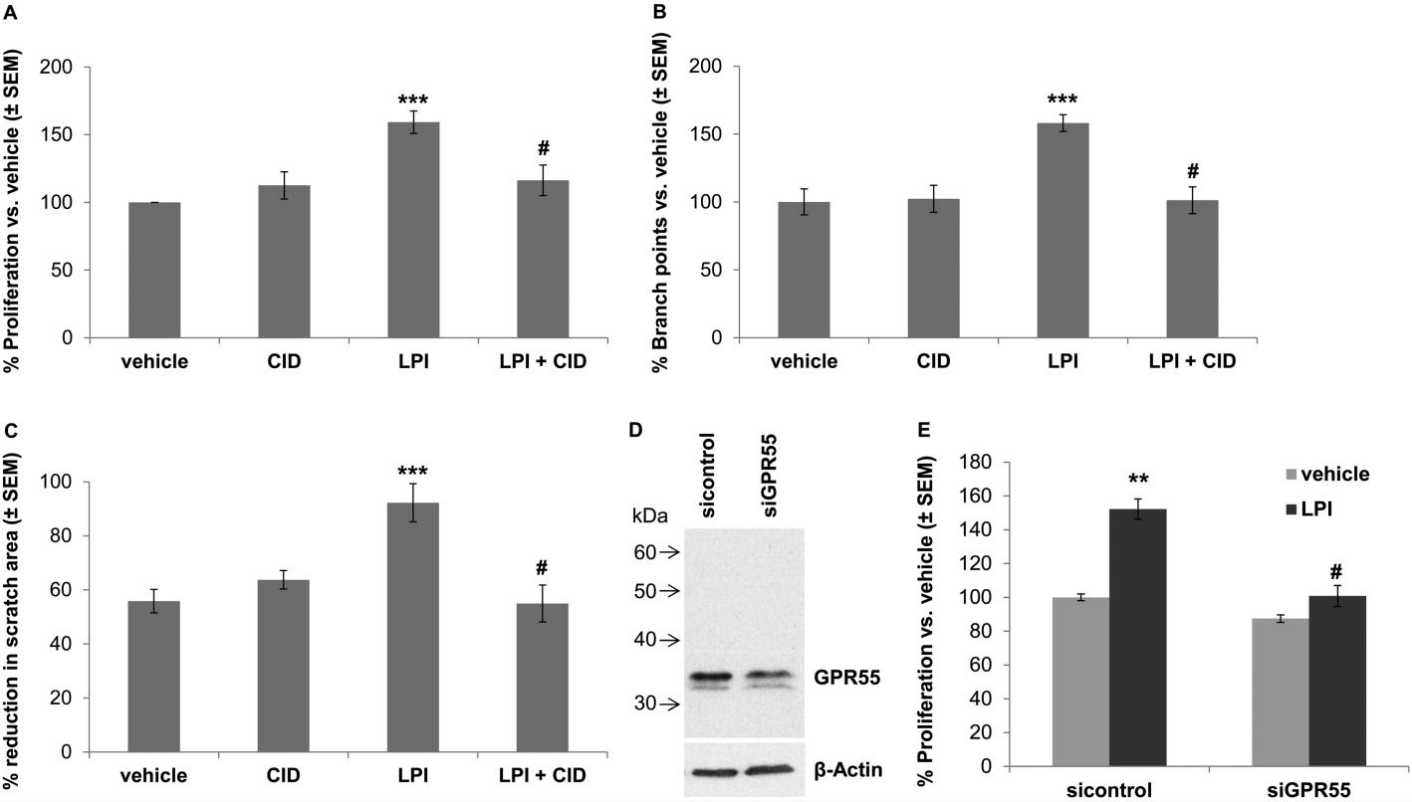
Pharmacological and siRNA inhibition of GPR55 prevents LPI-induced angiogenic activity of ECFCs *in vitro*. (A–C) Effect of vehicle, GPR55 inhibitor CID16020046 (20 μM; CID), LPI (10 μM) or LPI + CID on neonatal ECFC. (A) proliferation, shown in % as compared with vehicle control after 48 h *in vitro* proliferation assay. (B) Branch point formation, shown in % as compared with vehicle control in an *in vitro* angiogenesis assay after 16 h. (C) Closure of *in vitro* endothelial scratch area, shown in % as compared with vehicle control after 16 h. (D) Western blot analysis of GPR55 expression and β-actin in whole cell lysates of ECFCs transfected with control siRNA (sicontrol) or four selective siRNAs against GPR55 (siGPR55). (E) Proliferation increase of ECFCs transfected with control siRNA (sicontrol) or four selective siRNAs against GPR55 (siGPR55) in response to vehicle or 10 μM LPI (48 h). All *n* = 9; **P < 0.01, significantly different from vehicle sicontrol; ^#^*P* < 0.001, significantly different from LPI-treated sicontrol ECFCs. anova followed by Bonferroni test.

**Figure 4:**
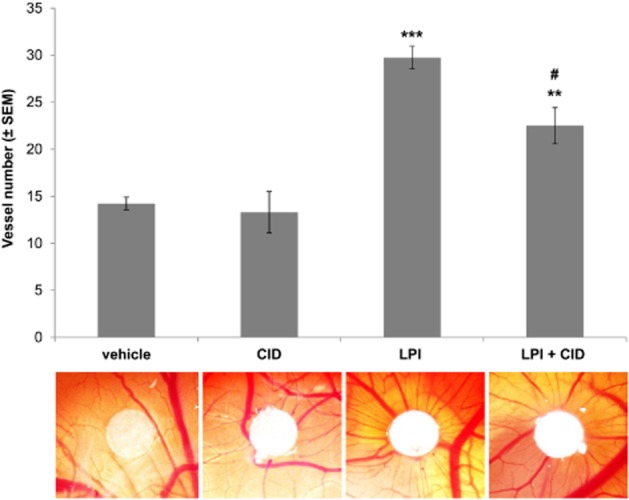
Pharmacological inhibition of GPR55 prevents LPI-induced angiogenesis in an *in vivo* chicken CAM assay. Quantification (by ImageJ) of vessel numbers around white filter paper in an *in vivo* CAM assay after 72 h. Filter papers were loaded with vehicle, 20 μM GPR55 inhibitor CID16020046 (CID), 10 μM LPI or both. Images are respective representative macroscopic pictures. *n* = 6–9; ***P < 0.001; **P < 0.01, significantly different from vehicle control; ^#^*P* < 0.001, significantly different from LPI treatment. anova followed by Bonferroni test.

To identify a pharmacological inhibitor of LPI-mediated pro-angiogenesis, we tested specific antagonists of known LPI receptors such as the CB_1_, CB_2_ recptors and GPR 55 (Pineiro and Falasca, 2012). The GPR55 antagonist CID16020046 (Kargl *et al*., 2013) decreased LPI-induced ECFC proliferation in a concentration-dependent manner with an IC_50_ of 17.9 (17.3–18.5) μM (Supporting Information Fig. S3a). LPI-stimulated ECFC proliferation was most effectively inhibited with a CID16020046 concentration of 20 μM, without affecting basal ECFC proliferation (Figure 3A). In contrast, the LPI-stimulated effect was not significantly inhibited by addition of the CB_1_ receptor antagonist/GPR55 agonist (AM251) or by antagonism of CB_2_ receptors with SR144528 (Supporting Information Fig. S3b). Furthermore, CID16020046 totally suppressed the LPI-induced network formation (Figure 3B) and endothelial wound healing (Figure 3C), without affecting the basal angiogenic capacity of endothelial cells. To confirm that LPI activity was GPR55 dependent, GPR55 was genetically knocked down with a mix of four validated siRNAs in ECFCs (Figure 3D). In response to LPI, siGPR55-ECFCs showed significantly reduced proliferation as compared with ECFCs transfected with control siRNA (Figure 3E). Simultaneous treatment with the GPR55 inhibitor CID16020046 significantly reduced the LPI-stimulated angiogenesis in the *in vivo* CAM model (Figure 4). Neither CID16020046 nor silencing of GPR55 significantly affected basal angiogenic activities of ECFCs *in vitro* nor angiogenesis in the CAM assay *in vivo* (Figures 3 and 4; Supporting Information Fig. S3). Altogether, these results demonstrate that exogenous LPI stimulates the pro-angiogenic capacity of ECFCs *in vitro* and angiogenesis *in vivo* in a specifically GPR55-dependent manner.

### LPI/GPR55 stimulates ERK1/2 and p38 activation

**Figure 5:**
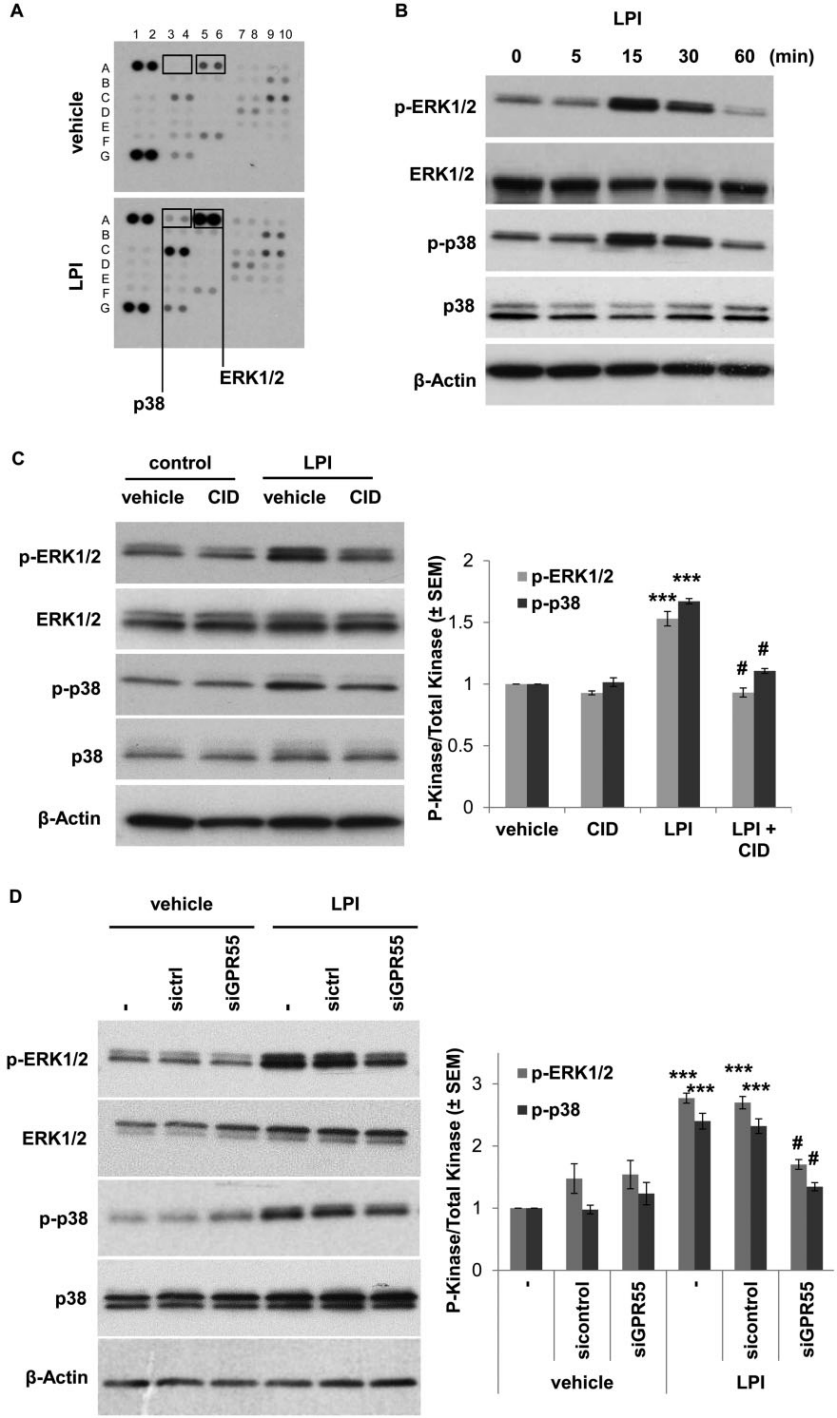
LPI-induced ERK1/2 and p38 phosphorylation is GPR55 dependent. (A) Human phospho-kinase array of whole neonatal-ECFC lysates after 15 min treatment with vehicle or 10 μM LPI. Pixel intensity, quantified by ImageJ, revealed an LPI-induced ERK1/2 and p38 phosphorylation. (B) Western blot analysis of total ECFC lysates after 0, 5, 15 and 30 min of 10 μM LPI treatment. Blots were probed with antibody against total or phosphorylated (p) ERK1/2 or p38 or β-actin. (C) Western blot analysis of total and phosphorylated ERK1/2 and p38 phosphorylation in ECFC lysates after 15 min treatment with vehicle, 20 μM CID16020046 (CID), 10 μM LPI or both. Quantification of the ratio of p-ERK1/2 or p-p38 normalized to β-actin immunostaining. *n* = 6; ***P < 0.001, significantly different from vehicle control; ^#^*P* < 0.001, significantly different from LPI-treated ECFCs. anova followed by Bonferroni test. (D) Western blot analysis of ERK1/2 and p38 phosphorylation in untreated (−) ECFCs or after transfection with control siRNA (sicontrol) or siRNA against GPR55 (siGPR55) in response to vehicle or 10 μM LPI (15 min). Quantification of the ratio of p-ERK1/2 or p-p38 normalized to β-actin immunostaining. *n* = 6; ***P < 0.001, significantly different from untreated vehicle control; ^#^*P* < 0.001, significantly different from LPI-treated sicontrol ECFCs. anova followed by Bonferroni test.

**Figure 6:**
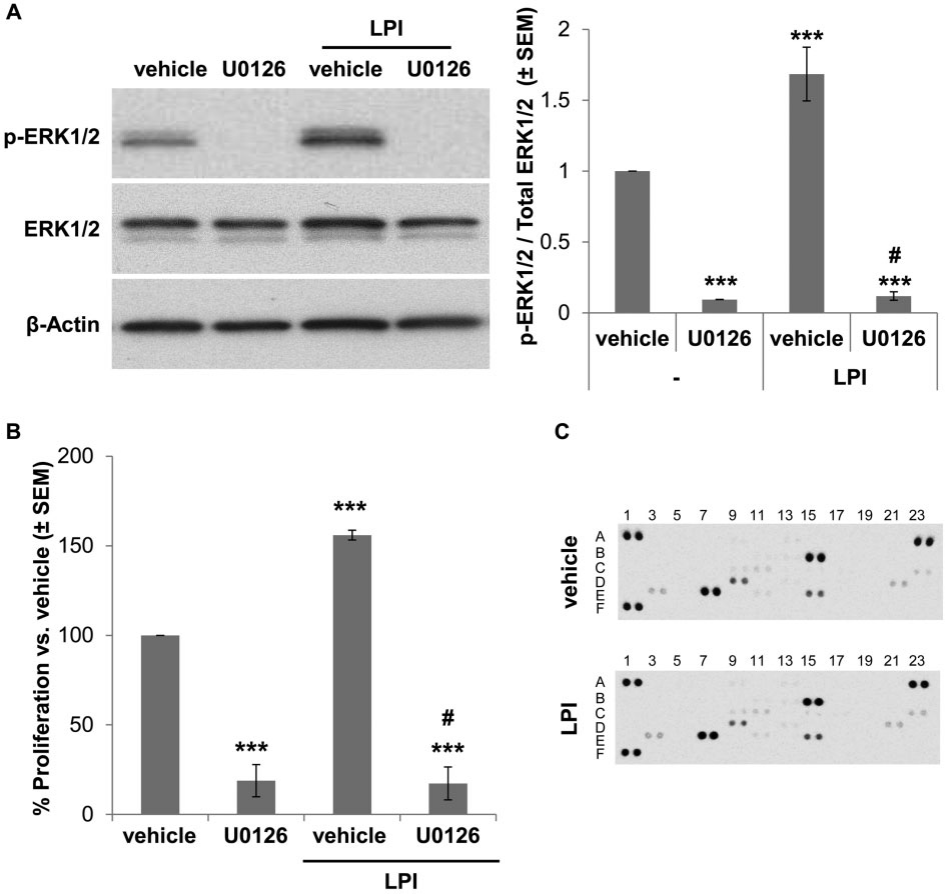
LPI-induced ECFC proliferation increase is blocked by ERK1/2-inhibitor U0126. (A) Western blot analysis of ERK1/2 phosphorylation in ECFC lysates after 30 min pre-incubation with vehicle or 10 μM U0126 and 15 min exposure to 10 μM LPI. Quantification of the ratio of p-ERK1/2 normalized to β-actin immunostaining. *n* = 6; ***P < 0.001, significantly different from vehicle control; ^#^*P* < 0.001, significantly different from LPI-treated ECFCs. anova followed by Bonferroni test. (B) Proliferation increase of ECFCs treated with vehicle or U0126 (48 h). *n* = 6; ***P < 0.001, significantly different from vehicle control; ^#^*P* < 0.001, significantly different from LPI-treated ECFCs. anova followed by Bonferroni test. (C) Proteome profiler human angiogenesis array of neonatal-ECFC supernatants after 24 h treatment with vehicle or 10 μM LPI. Pixel intensity was quantified by ImageJ.

A human phospho-kinase array was used to investigate the molecular mechanisms underlying LPI/GPR55-mediated angiogenesis. Of the various phospho-proteins in the array, LPI significantly induced phosphorylation of only ERK1/2 and p38 in ECFCs (Figure 5A and Supporting Information Table S1). Western blot analysis confirmed a time-dependent activation of ERK1/2 and p38 by 10 μM LPI (Figure 5B) but not the potential involvement of CREB (cAMP response element-binding protein) (data not shown). Pharmacological inhibition of GPR55 by 20 μM CID16020046 significantly reduced LPI-induced ERK1/2 and p38 phosphorylation (Figure 5C). To confirm the GPR55-dependent activation of ERK1/2 and p38 by LPI, GPR55 was silenced by siRNA (Figure 5D). Compared with control siRNA, knock-down of GPR55 suppressed the LPI-stimulated ERK1/2 and p38 phosphorylation (Figure 5D). Moreover, ECFCs pretreated with the well-established and highly selective MEK1/MEK2 inhibitor U0126 (10 μM) (Favata *et al*., 1998), which blocks downstream activation of ERK1/2, eliminated ERK1/2 basal phosphorylation without altering total ERK1/2 amounts (Figure 6A). However, LPI no longer induced ERK1/2 phosphorylation (Figure 6A). Furthermore, ERK1/2 inhibition blocked normal ECFC proliferation, without reducing the initial cell number, and prevented the LPI-induced ECFC proliferation, indicating a crucial role for ERK1/2 during endothelial cell proliferation (Figure 6B). Together, these results suggest that LPI induces GPR55-dependent activation of ERK1/2 and thus leads to increased angiogenesis.

We further investigated whether the observed pro-angiogenic effect of LPI relies on an LPI-induced up-regulation of angiogenesis-related proteins and thereby indirectly leads to an autocrine angiogenic feedback loop with an activation of ERK1/2 and p38. A proteome profiler human angiogenesis array revealed that LPI did not lead to an altered production of any of the 55 tested known angiogenesis-related proteins from ECFCs within 24 h (Figure 6C and Supporting Information Table S2).

## Discussion

Ovarian carcinomas are highly vascularized tumours (Schoell *et al*., 1997; Domcke *et al*., 2013; Sinha *et al*., 2013). LPI produced and secreted by ovarian and prostate cancer cells has been shown to induce a GPR55-dependent autocrine loop regulating cancer growth (Pineiro *et al*., 2011). Although the intracellular effect of LPI and its receptor GPR55 has been extensively studied, to this date a causative role of LPI/GPR55 in (tumour) angiogenesis *in vivo* and its underlying molecular mechanism in endothelial cells remains uncharacterized. In the present study, we demonstrated that ovarian cancer cells produced and secreted LPI which stimulated ECFC angiogenic potential *in vitro* and *in vivo* angiogenesis in the CAM in a GPR55-dependent manner via activation of the MAPK pathway.

The OVCAR-3, OVCAR-5 and COV-362 cell lines were derived from patients with high-grade serious ovarian cancer and formed highly vascularized tumours (Godwin *et al*., 1992; Domcke *et al*., 2013; Sinha *et al*., 2013). In the present study, we have demonstrated that these ovarian carcinoma cell lines secrete LPI and induce *in vivo* CAM angiogenesis in a GPR55-dependent manner. Even though other mediators, as VEGF, are most likely also involved in this process, the fact that blocking of GPR55 inhibits LPI-induced vessel number suggests that this is a LPI-mediated event. We therefore hypothesized that LPI secreted by ovarian carcinomas stimulates endothelial pro-angiogenic activities (i.e. proliferation, migration, network formation) and increases angiogenesis. Very few reports have been published yet on the effect of LPI on endothelial cell angiogenic activity (Pineiro and Falasca, 2012). It has been shown that LPI induces *in vitro* proliferation of human microvascular endothelial cells (HMVECs) (Zhang *et al*., 2010). Effects on endothelial cell motility have been studied but with contradictory results (Murugesan and Fox, 1996; Kargl *et al*., 2013). Murugesan and Fox (1996) showed an LPI-induced decrease of dermal-derived HMVEC migration, whereas Kargl *et al*. (2013) showed a stimulatory effect of LPI on motility of lung-derived HMVECs. These differing results might be due to the endothelial cells being isolated from different vascular beds. We investigated the effect of LPI on human ECFCs *in vitro*, cells with robust proliferative and vasculogenic capabilities (Yoder *et al*., 2007). We found that LPI is a potent stimulant for ECFC proliferation, migration and network formation *in vitro* and is an effective pro-angiogenic factor in the *in vivo* CAM assay. LPI stimulated ECFC proliferation at low concentrations (about 1 nM) and reached its maximum pro-proliferative potential at 10 μM. The stimulatory effect of LPI on ECFC proliferation was confirmed in other endothelial cell sources as well, including human adult peripheral ECFCs and HUVECs. It would be worthwhile to investigate the effects of LPI on additional endothelial cell sources and also on non-endothelial cells.

The *in vitro* and *in vivo* stimulatory effects of LPI were reduced by pharmacological (CID16020046) and genetic inhibition (siRNA) of GPR55. LPI specifically activates GPR55 but not CB_1_ or CB_2_ receptors (Bondarenko *et al*., 2010; Kargl *et al*., 2013; Liu *et al*., 2015). Consistent with this specificity, inhibition of these CB receptors did not significantly affect the LPI-induced proliferation of ECFCs. Together, these results confirm that the LPI-mediated effects on angiogenesis *in vitro* and *in vivo* are regulated by GPR55. However, LPI-induced ECFC proliferation and *in vivo* angiogenesis could not be eliminated completely by pharmacological or genetic GPR55 inhibition. This is in accordance with previous reports of an additional GPR55-independent endothelial cell depolarization by LPI (Bondarenko *et al*., 2010; 2011a,b[Bibr b8],[Bibr b9]).

Mechanistically, we showed that LPI stimulated a GPR55-dependent phosphorylation of ERK1/2 and p38. Ovarian cancer cell supernatants also significantly stimulated ERK1/2 and p38 according to the lower LPI concentration. In the pro-angiogenic signalling cascade ERK1/2 is a well-established mediator of proliferation (Zhang and Liu, 2002), while p38 has been shown to regulate actin reorganization and thereby cell migration (Rousseau *et al*., 1997; Lamalice *et al*., 2007). Therefore, ERK1/2 inhibition by U0126 blocked LPI-induced proliferation of ECFCs. ECFCs showed basal levels of ERK1/2 indicating an essential role of ERK1/2 during normal endothelial cell proliferation. This could explain why inhibition of ERK1/2 also inhibited normal ECFC proliferation. Our results suggest a crucial role of the MAPK pathway also during LPI-induced angiogenesis. The finding that LPI does not alter the basal production of the tested angiogenesis-related proteins, as VEGF, suggests that LPI does not activate an autocrine loop. Nonetheless, our data show that LPI significantly stimulates a pro-angiogenic response of endothelial cells. This leaves open to question whether the pro-angiogenic properties of LPI are due to a facilitated angiogenic response to the basal levels of growth factors produced by ECFCs or if this process involves other yet unknown mediators.

Interestingly, neither the GPR55 inhibitor CID16020046 nor silencing of GPR55 with siRNAs had a significant effect on any of the tested basal angiogenic functions of endothelial cells. This suggests that normal blood vessels would not be inhibited when applying CID16020046, possibly only the LPI-induced angiogenesis as in ovarian carcinoma. Therefore, CID16020046 could be of potential interest as an anti-(ovarian) cancer drug. Although beyond the scope of this study, it would be of interest to study the effect of the pharmacological GPR55 inhibitor CID16020046 on ovarian tumour size and vascularity in mice, and furthermore to investigate the involvement of ovarian tumour angiogenesis in GPR55 knockout mice *in vivo*. Our hypothesis is that LPI is an endogenous factor secreted upon a pathological event (e.g. after ischaemia, wound healing or from cancer cells) (Pineiro *et al*., 2011; Pineiro and Falasca, 2012), leading to increased angiogenesis. Further investigation of the physiological and pathological circumstances leading to LPI production by different cell sources would also be of interest in determining the physiological role of LPI and GPR55 inhibition *in vivo*.

In summary, our results show that LPI is a (ovarian) tumour-derived pro-angiogenic factor that acts through GPR55-dependent activation of ERK1/2 and p38 in endothelial cells. Our data suggest the LPI/GPR55 axis may be a significant target for the development of pro- and anti-angiogenic therapies. Further, we propose that the GPR55 antagonism (e.g. by CID16020046) could be of potential interest to develop an anti-tumour angiogenesis treatment (e.g. for patients with ovarian carcinoma).

## Abbreviations

bFGF: basic fibroblast growth factor
CID16020046: 4-[4-(3-hydroxyphenyl)-3-(4-methylphenyl)-6-oxo-1H,4H,5H,6H-pyrrolo [3,4-c] pyrazol-5-yl] benzoic acid
ECFC: endothelial colony-forming cell
LPI: L-α-lysophosphatidylinositol

## Supporting Information

**Figure S1** Phenotypical characterization of endothelial colony-forming progenitor cells (ECFCs). **Figure S2** LPI concentration dependently stimulates proliferation in different endothelial cell sources. **Figure S3** LPI-induced ECFC proliferation is GPR55 dependent. **Table S1** LPI activates ERK1/2 and p38. **Table S2** LPI effect on human proteome profile of angiogenesis-related proteins.
