# Supplementary material for: The GPR 55 agonist, L-α-lysophosphatidylinositol, mediates ovarian carcinoma cell-induced angiogenesis
Source: Br J Pharmacol. 2015 Jun 26;172(16):4107–18. doi: 10.1111/bph.13196 (PMC4543616; doi:10.1111/bph.13196)
Supplement: Supplementary file 1 [file bph0172-4107-sd1.pdf]

Supplementary Fig. S1 **Hofmann et al.**

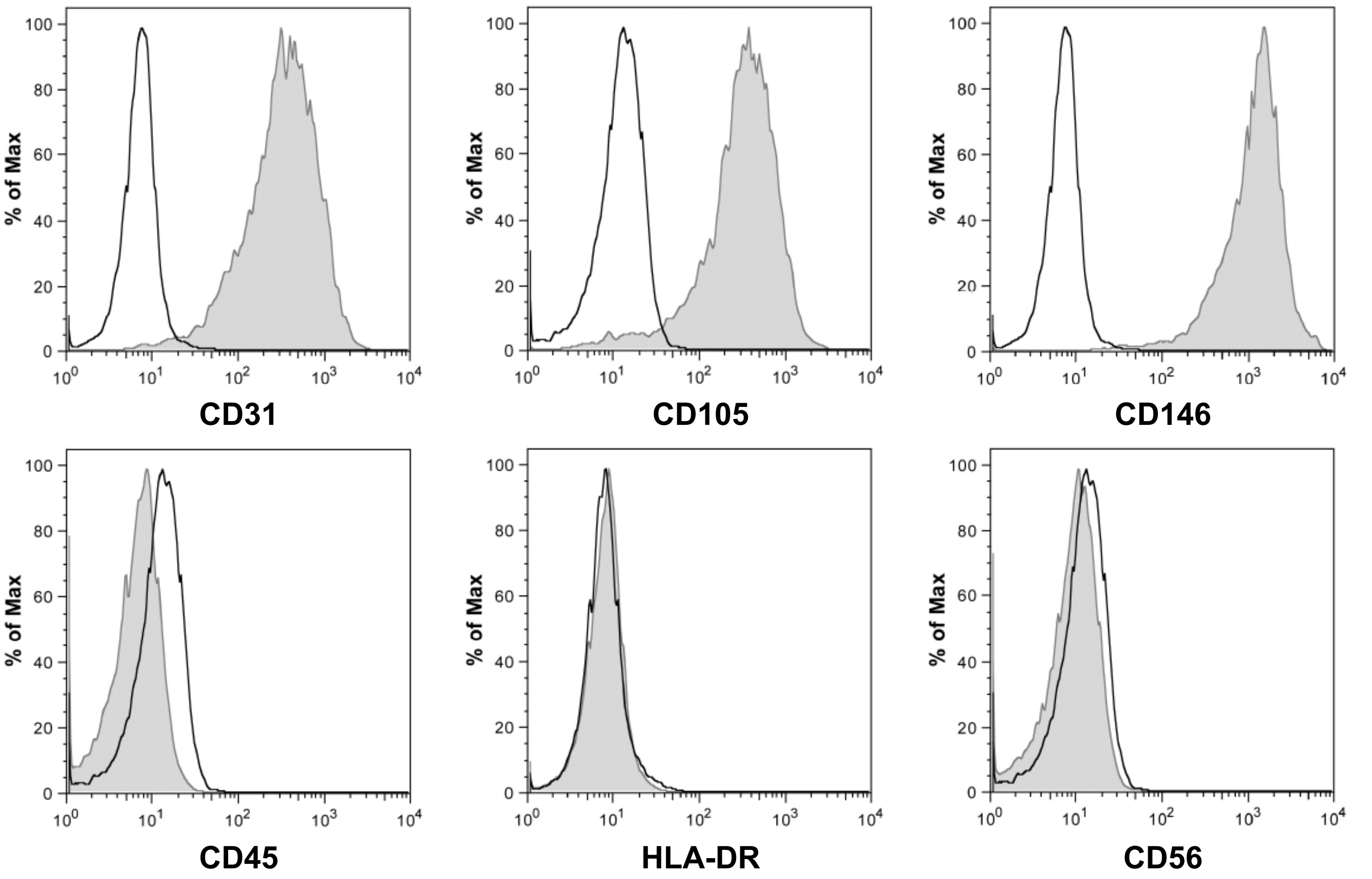

Supplementary Figure S1. Phenotypical characterization of endothelial colony-forming progenitor cells (ECFCs). Representative flow cytometry histograms of ECFCs showing reactivity with typical endothelial cell-expressed molecules CD31, CD105 and CD146 (right-shifted filled gray curves compared with black lined open curves of the appropriate isotype controls) and lack of reactivity with hematopoietic (CD45) and human leukocyte antigen class II type DR (HLA-DR) markers.

Supplementary Fig. S2 **Hofmann et al.**

A

B

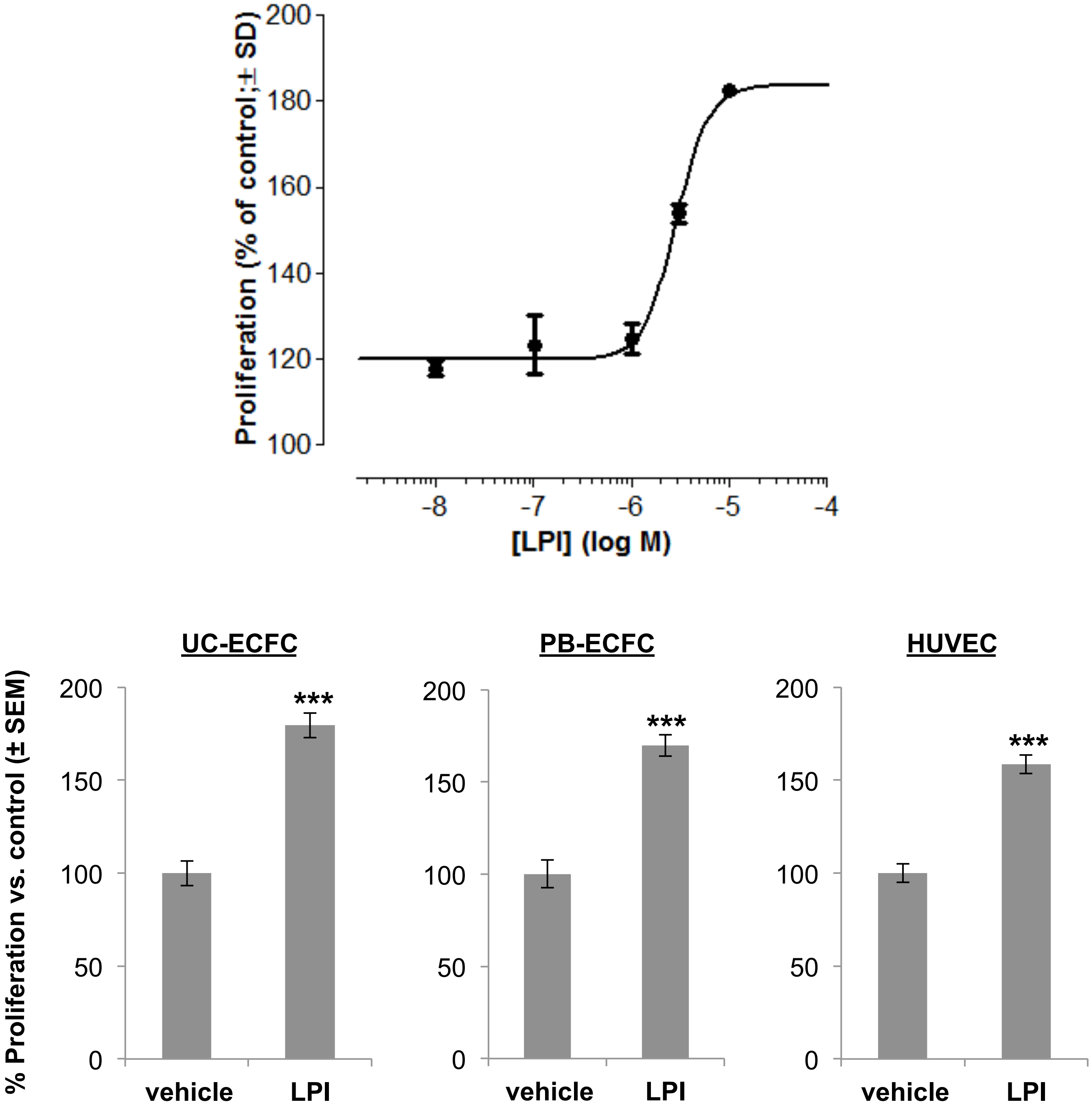

Supplementary Figure S2. LPI concentration-dependently stimulates proliferation in different endothelial cell sources. (**A**) Effect of different concentrations (0.01, 0.1, 1, 3, 10 µM) lysophosphatidylinositol (LPI) on proliferation of neonatal-endothelial colony forming progenitor cell (ECFC). Values represent % proliferation increase versus vehicle control. (**B**) Proliferation increase (x 10^3) of umbilical cord-derived ECFCs (UC-ECFC), human adult peripheral blood ECFCs (PB-ECFC) or human umbilical vein endothelial cells (HUVECs) after 48 hours treatment with vehicle of 10 µM LPI. n=5; ***p<0.001, Student t-test.

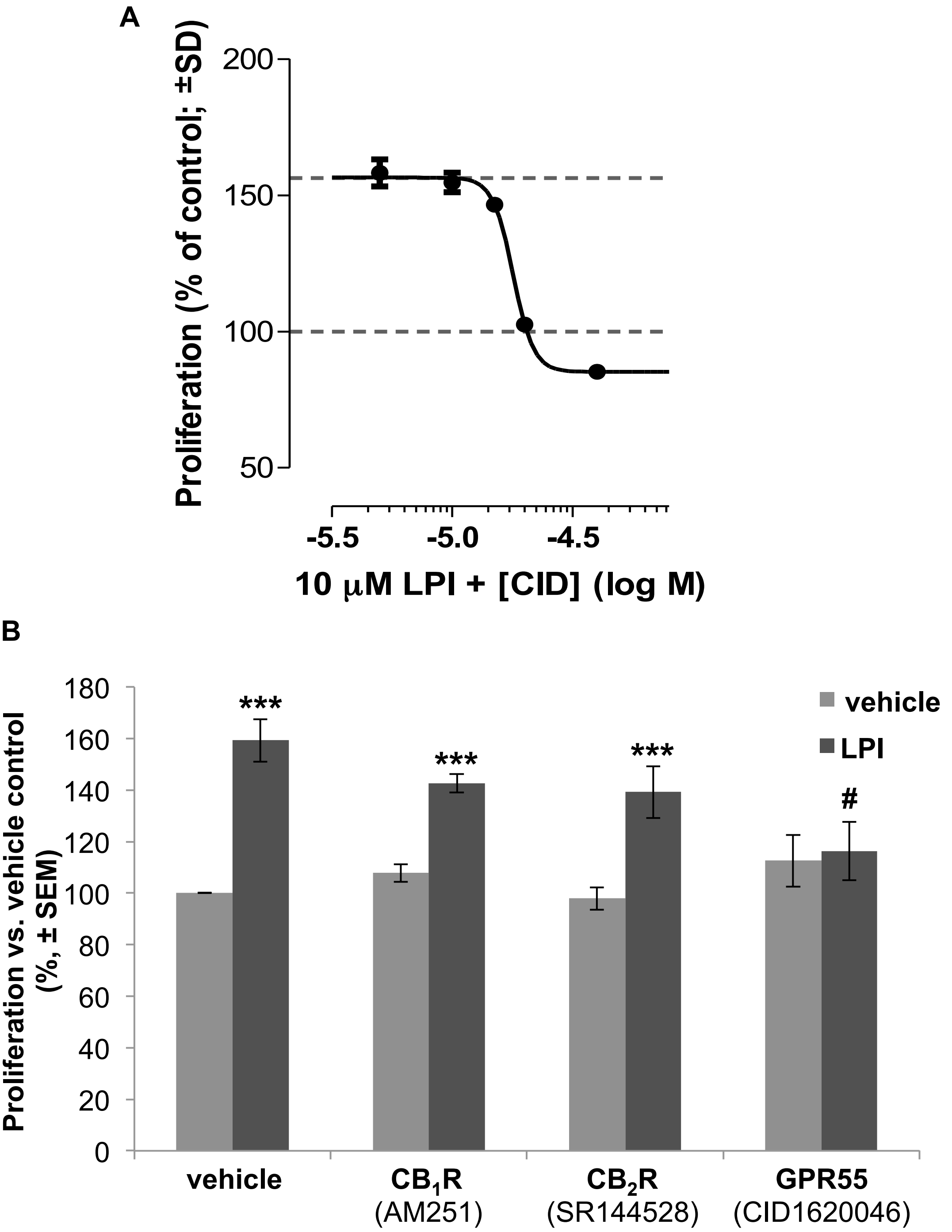

Supplementary Figure S3. LPI-induced ECFC proliferation is GPR55-dependent. (**A**) Effect of different concentrations (5, 10, 15, 20, 40 μM) of the GPR55-inhibitor CID16020046 (CID) on 10 μM LPI stimulated proliferation of neonatal-endothelial colony forming progenitor cell (ECFC). Values represent % proliferation increase versus vehicle control. Lower dotted line represents value of vehicle ECFCs, upper dotted line represents value of 10 μM LPI treated ECFCs. (**B**) Effect of vehicle, cannabinoid receptor 1 (CB_1R)-inhibitor AM251, CB_2R-inhibitor SR144528 or GPR55-inhibitor CID16020046 on ECFC proliferation with or without 10 μM LPI treatment. Values are represented as compared to vehicle control. n=9;***p<0.001 as compared to vehicle control; $^{\#}$p<0.001 as compared to LPI treated ECFCs. ANOVA followed by Bonferroni test.

Supplementary Table 1 Hofmann et al.

		Phospho-antibody	LPI vs. vehicle (ratio)
A	1,2	Reference spot	0.95
	3,4	p38	10.95
	5,6	ERK1/2	3.39
	7,8	JNK1/2/3	1.95
	9,10	GSK-3α/β	0.74
B	3,4	EGF-R	0.82
	5,6	MSK1/2	1.27
	7,8	AMPKα1	0.49
	9,10	Akt1/2/3 (S473)	0.90
C	1,2	mTOR	0.85
	3,4	CREB	1.92
	5,6	HSP27	0.73
	7,8	AMPKα2	0.68
	9,10	β-Catenin	0.59
D	1,2	Src	0.51
	3,4	Lyn	0.74
	5,6	Lck	0.67
	7,8	STAT2	0.59
	9,10	STAT5α	0.47
E	1,2	Fyn	0.72
	3,4	Yes	0.82
	5,6	Fgr	1.11
	7,8	STAT6	1.06
	9,10	STAT5β	0.79
F	1,2	Hck	1.88
	3,4	Chk-2	1.11
	5,6	FAK	1.07
	7,8	PDGFRb	0.74
	9,10	STAT5α/β	0.93
G	1,2	Reference Spot	0.89
	3,4	PRAS40	1.74
	9,10	Negative ctrl	0.00

Supplementary Table 1. LPI activates ERK1/2 and p38. Human phospho-kinase array of whole neonatal-ECFC lysates after 15 minutes treatment with vehicle or 10 µM lysophosphatidylinositol (LPI). Pixel intensity was quantified by ImageJ after background subtraction ratio of LPI treated ECFCs vs. vehicle treated ECFCs was evaluated. Ratio increase was considered significant at a ratio above 2.0.

Supplementary Table 2 Hofmann et al.

			LPI vs. vehicle (ratio)
A	1,2	Reference spot	0,95
	5,6	Activin A	0,93
	7,8	ADAMTS-1	0,73
	9,10	Angiogenin	0,97
	11,12	Angiopoietin-1	0,99
	13,14	Angiopoietin-2	0,86
	15,16	Angiostatin/Plasminogen	0,99
	17,18	Amphiregulin	0,85
	19,2	Artemin	0,82
	23,24	Reference spot	0,95
B	1,2	Coagulation Factor III	0,78
	3,4	CXCL16	0,75
	5,6	DPPIV	0,74
	7,8	EGF	0,75
	9,10	EG-VEGF	0,76
	11,12	Endoglin	0,75
	13,14	Endostatin/Collagen XVIII	0,85
	15,16	Endothelin-1	0,88
	17,18	FGF acidic	0,81
	19,2	FGF basic	0,74
	21,22	FGF-4	0,83
	23,24	FGF-7	0,77
C	1,2	GDNF	0,56
	3,4	GM-CSF	0,54
	5,6	HB-EGF	0,80
	7,8	HGF	0,61
	9,10	IGFBP-1	0,73
	11,12	IGFBP-2	0,68
	13,14	IGFBP-3	0,59
	15,16	IL-1b	0,85
	17,18	IL-8	0,93
	19,2	LAP (TGF-b1)	0,69
	21,22	Leptin	0,47
	23,24	MCP-1	1,03
D	1,2	MIP-1a	1,11
	3,4	MMP-8	1,03
	5,6	MMP-9	1,13
	7,8	NRG1-b1	1,03
	9,10	Pentraxin 3	1,05
	11,12	PD-ECGF	1,13
	13,14	PDGF-AA	1,44
	15,16	PDGF-AB/PDGF-BB	1,12
	17,18	Persephin	0,95
	19,2	CXCL4	1,01
	21,22	PIGF	1,00
	23,24	Prolactin	0,94
E	1,2	Serpin B5	0,70
	3,4	Serpin E1	1,00
	5,6	Serpin F1	0,83
	7,8	TIMP-1	0,90
	9,10	TIMP-4	0,77
	11,12	Thrombospondin-1	0,70
	13,14	Thrombospondin-2	0,60
	15,16	u PA	0,91
	17,18	Vasohibin	0,96
	19,2	VEGF	0,84
	21,22	VEGF-C	0,73

Supplementary Table 2. LPI effect on human proteome profile of angiogenesis related proteins. Human angiogenesis proteome array of neonatal-ECFC supernatants after 24 hour treatment with vehicle or 10 µM lysophosphatidylinositol (LPI). Pixel intensity was quantified by ImageJ after background subtraction ratio of LPI treated ECFCs vs. vehicle treated ECFCs was evaluated. Ratio increase was considered significant at a ratio above 2.0.
